# Effects of echinocandin preparations on adult rat ventricular cardiomyocytes: Preliminary results of an in vitro study

**DOI:** 10.1007/s00101-014-2289-8

**Published:** 2014-02-07

**Authors:** C. Arens, F. Uhle, M. Wolff, R. Röhrig, C. Koch, A. Schulte, S. Weiterer, M. Henrich, M.A. Weigand, K.-D. Schlüter, C. Lichtenstern

**Affiliations:** 1 Department of Anesthesiology and Intensive Care Medicine, University Hospital of Gießen and Marburg, Campus Gießen, Rudolf-Buchheim-Str. 7, 35392 Gießen, Germany; 2 Department of Physiology, Justus-Liebig-University of Giessen, Gießen, Germany

**Keywords:** Antifungal agents, Echinocandins, Adverse drug reaction, Cardiomyopathy, Organ failure, Antimykotika, Echinocandine, Nebenwirkung, Kardiomyopathie, Organversagen

## Abstract

**Background:**

*Candida* infections represent a relevant risk for patients in intensive care units resulting in increased mortality. Echinocandins have become the agents of choice for early and specific antifungal treatment in critically ill patients. Due to cardiac effects following echinocandin administration seen in intensive care unit (ICU) patients the in vitro effects of echinocandins and fluconazole on isolated cardiomyocytes of the rat were examined.

**Aim:**

The study was designed to investigate a possible impact of echinocandins and fluconazole in clinically relevant concentrations on the in vitro contractile responsiveness and shape of isolated rat cardiomyocytes.

**Material and methods:**

Ventricular cardiomyocytes were isolated from Lewis rats. Cardiomyocytes were cultured in the presence of all licensed echinocandin preparations and fluconazol at concentrations of 0 (control), 0.1, 1, 3.3, 10, 33 and 100 μg/ml for 90 min. Cells were stimulated by biphasic electrical stimuli and contractile responsiveness was measured as shortening amplitude. Additionally, the ratio of rod-shaped to round cells was determined.

**Results:**

Anidulafungin concentrations of 3.3 and 10 μg/ml caused a significant increase in contractile responsiveness, caspofungin showed a significant decrease at 10 μg/ml and micafungin concentrations of 3.3–33 μg/ml led to a significant increase in cell shortening. Measurement was not possible at 33 μg/ml for anidulafungin and caspofungin and at 100 μg/ml for all echinocandins due to a majority of round-shaped, non-contracting cardiomyocytes. Fluconazole showed no significant effect on cell shortening at all concentrations tested. For the three echinocandins the ratio of round-shaped, non-contracting versus rod-shaped normal contracting cardiomyocytes increased in a dose-dependent manner.

**Conclusions:**

Echinocandins impact the in vitro contractility of isolated cardiomyocytes of rats. This observation could be of great interest in the context of antifungal treatment.

## Abstract (de)

**Hintergrund:**

*Candida*-Infektionen stellen ein relevantes Risiko für Patienten einer Intensivstation dar und führen zu einer erhöhten Mortalität. Echinocandine sind mittlerweile Mittel der Wahl für die frühzeitige und spezifische antimykotische Behandlung kritisch kranker Patienten. Aufgrund kardialer Wirkungen nach Echinocandingabe bei den Intensivpatienten der Autoren wurden die In-vitro-Wirkungen von Echinocandinen und Fluconazol auf isolierte Kardiomyozyten der Ratte untersucht.

**Ziel der Arbeit:**

Die Studie wurde durchgeführt, um den möglichen Einfluss von Echinocandinen und Fluconazol in klinisch relevanten Konzentrationen auf die In-vitro-Kontraktilität und die Form isolierter Rattenkardiomyozyten zu bestimmen.

**Material und Methoden:**

Ventrikuläre Kardiomyozyten wurden aus Lewis-Ratten isoliert. Die Kardiomyozyten wurden mit sämtlichen zugelassenen Echinocandinpräparaten und Fluconazol in den Konzentrationen von 0 (Kontrolle); 0,1; 3,3; 10; 33 und 100 μg/ml über 90 min kultiviert. Mit biphasischen elektrischen Reizen erfolgte die Stimulation der Zellen, und die Kontraktilität wurde als Verkürzung der Amplitude gemessen. Außerdem wurde das Verhältnis stäbchenförmiger Zellen zu runden Zellen ermittelt.

**Ergebnisse:**

Eine Anidulafunginkonzentration von 3,3 sowie von 10 μg/ml verursachte einen signifikanten Anstieg der Kontraktilität; bei Caspofungin zeigte sich eine signifikante Verminderung bei 10 μg/ml; und Micafunginkonzentrationen von 3,3–33 μg/ml führten zu einem signifikanten Anstieg der Zellverkürzung. Aufgrund einer Mehrzahl von runden, nichtkontraktilen Kardiomyozyten war die Messung bei einer Konzentration von 33 μg/ml Anidulafungin und Caspofungin sowie für alle Echinocandine bei 100 μg/ml nicht möglich. Bei Fluconazol fand sich in allen analysierten Konzentrationen keine signifikante Wirkung auf die Zellverkürzung. Bei den 3 Echinocandinen stieg das Verhältnis runder, nichtkontraktiler Zellen gegenüber den stäbchenförmigen, normalkontraktilen Kardiomyozyten dosisabhängig an.

**Schlussfolgerung:**

Echinocandine beeinflussen die In-vitro-Kontraktilität isolierter Kardiomyozyten von Ratten. Diese Beobachtung könnte im Rahmen einer antimykotischen Behandlung von hohem Interesse sein.

## Background

*Candida* infections represent a relevant risk for patients in intensive care units (ICU; [1, 2]) resulting in increased mortality [3, 4, 5]. Adequate antifungal treatment is essential in patients with sepsis to prevent irreversible damage due to microbial load, systemic inflammation and organ failure [6].

The echinocandins are large (molecular weight ~ 1200 kDa) semisynthetic cyclic hexapeptides derived from various natural fungal products [7, 8]. The lipophilic side chain of the echinocandins intercalates with the phospholipid bilayer of the fungal cell membrane where it serves as a non-competitive inhibitor of β-1,3-D-glucan synthase. Deficiency of β-1,3-D-glucan in the fungal cell wall results in osmotic instability and fungal cell lysis. The echinocandins have become the antifungal agents of first choice due to the excellent activity against most *Candida* strains and the favorable safety profile. Guidelines recommend echinocandins as primary therapy for all forms of candidiasis, especially in severely ill patients with organ dysfunction [9, 10]. Development of septic cardiomyopathy reflects a crucial pathogenic part of hemodynamic instability in septic shock which aggravates tissue hypoperfusion and organ failure [11]. Due to cardiac effects following echinocandin administration seen in ICU patients [12] the in vitro effects of echinocandins and fluconazole in clinically relevant concentrations on isolated cardiomyocytes of the rat were examined.

## Material and methods

### Material

Stock solutions of the three echinocandins anidulafungin (Ecalta®, Pfizer, Illertissen, Germany), micafungin (Mycamine®, Astellas Pharma Europe, Leiden, The Netherlands) and caspofungin (Cancidas®, Merck Sharp & Dohme, Hertfordshire, UK) as well as fluconazole (Diflucan®, Pfizer) were made by reconstitution of licensed preparations according to the product information. These were diluted directly before use with distilled water and 10 µl of this dilution was added to 1 ml culture medium resulting in concentrations of 0.1–100 µg/ml. Control cultures were treated with distilled water only.

### Isolation of cardiomyocytes

Animal experiments (sacrificing animals and organ extraction) were performed with approval by the animal welfare officer of the Faculty of Medicine of the Justus-Liebig University Giessen and in accordance with Federation of European Laboratory Animal Science Associations (FELASA) guidelines. Ventricular heart muscle cells were isolated from Lewis rats in a standard procedure as described in greater detail previously [13, 14]. Briefly, hearts from Lewis rats (age 3–4 months) were excised under deep ether anesthesia and mounted on the cannula of a Langendorff perfusion system. Cardiomyocytes were isolated via perfusion with collagenase, followed by mincing, filtering and transfer to culture medium M199 supplemented with carnitine (2 mM), creatine (5 mM) and taurine (5 mM).

### Incubation

A time-effect curve was constructed to investigate the representative duration for echinocandin incubation. Cardiomyocytes were incubated with 10 μg/ml caspofungin for 15 min, 90 min and 4 h, respectively. Subsequently, experiments were performed after cardiomyocytes were cultured in the presence of all licensed echinocandin preparations at concentrations of 0 (control), 0.1, 1, 3.3, 10, 33, and 100 μg/ml for 90 min. For fluconazole experiments concentrations of 0 (control), 0.1, 1, 3.3, 10, 20, and 100 μg/ml were used. Due to the fact that fluconazole is supplied in a ready to use preparation (2 mg/ml), the highest achievable concentration in the experimental setup was 20 µg/ml. Therefore, 100 µg/ml was achieved by adding five times the volume (= 50 µl) compared to echinocandin preparations. To mimic plasma conditions two different methods of albumin incubation were used: the first series was performed by adding echinocandin preparations in culture medium containing 10 mg/ml albumin for 15 min followed by replacing the original culture medium by the culture medium-albumin-echinocandin mix and incubating the cells again for 90 min. An albumin concentration of 10 mg/ml was experimentally determined as the highest concentration not affecting cardiomyocyte viability and contractility. The second series was performed by applying echinocandin preparations in culture medium containing 10 mg/ml albumin that has already been in the culture dish. Each experiment was performed in duplicate and in a blinded manner.

### Contractility measurements

Cell contraction was investigated using a cell edge detection system as described previously [15]. Briefly, cells were stimulated by biphasic 50 V electrical stimuli of 0.5 ms duration via field stimulation by AgCl electrodes. Each cell was stimulated at 2 Hz for 1 min. Cell contraction was measured at 5 intervals of 15 s and the mean of these 4 measurements was used to define the contractile responsiveness of a given cell. Cell lengths were measured at a rate of 500 Hz via a line camera. Data are expressed as ΔL/L (%) in which the shortening amplitude (ΔL) is expressed as a percentage of the diastolic cell length (L). At least 37 randomly selected cells from 2 independent cardiomyocyte isolations were analyzed for each concentration and substance.

### Rod-shaped:round cell ratio

Photographs of cultured cardiomyocytes were taken with a BZ-800K microscope (Keyence, Neu-Isenburg, Germany). From each culture dish five photographs from separate sections were taken and the rod-shaped:round cell ratio was determined. On average approximately 400 cells were analyzed per condition from 6 culture dishes out of 2 preparations. Data were analyzed in a blinded manner.

### Statistical analysis

Results are expressed as mean ± standard deviation. Differences between groups were analyzed globally by one-way ANOVA, followed by Dunnet’s post-test to compare different treatment groups to controls. A value of *p* < 0.05 was regarded as significant. All analyses were done using GraphPad Prism version 5.04 for Mac (GraphPad Software, San Diego CA).

## Results

**Fig. 1:**
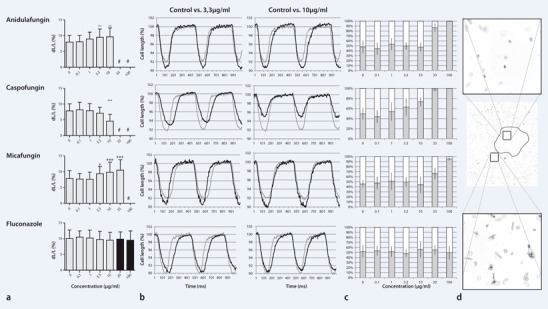
Effect of different antifungal preparations and concentrations on cellular parameters of isolated rat cardiomyocytes. **a** Contractility responsiveness measured as shortening ratio (dL/L). Graphs show mean values ± standard deviation. **b** Single cell observation of contractility at 3.3 and 10 µg/ml versus controls. **c** Rounded cells *(grey bars)* and rod-shaped cells *(white bars)* were counted. **d** Representive micrograph of culture dish following high-dose echinocandins given in culture medium containing 10 mg/ml albumin. *Middle picture*: overview, *top and bottom*: magnified areas of overview picture. *Black line* depicts limits of impact area

In the experiments anidulafungin concentrations of 3.3, and 10 μg/ml showed a significant increase of contractility responsiveness in contrast to 0.1 and 1 μg/ml which were not different compared to controls (Fig. 1 **a**,**b**; Tab. 1). For caspofungin cell shortening was not different compared to controls for 0.1–3.3 μg/ml; however, incubation with 10 μg/ml showed a significant decrease. Micafungin concentrations of 0.1 and 1 μg/ml showed no effect in contrast to controls while incubation with 3.3–33 μg/m led to a significant increase in cell shortening. Measurement was not possible at 33 μg/ml for anidulafungin and caspofungin and at 100 μg/ml for all echinocandins due to a majority of round-shaped, non-contracting cardiomyocytes. Fluconazole showed no significant effect on cell shortening in all analyzed concentrations.

**Tab. 1:** Values of mean contractility *(mean dL/L)*, standard deviation *(SD)*, number of cells *(N)* and significance compared to control for all concentrations and preparations

		Concentration (µg/ml])						
		Control	0.1	1	3.3	10	33	100
Anidulafungin	Mean dL/L	7.899	8.096	8.970	9.479	9.627		
	SD	2.101	1.964	2.164	2.646	2.800		
	N	54	54	44	54	37		
	Significance vs. control	ns	ns	ns	**	**		
Caspofungin	Mean dL/L	8.053	8.359	7.951	7.095	4.834		
	SD	1.923	2.372	2.191	1.944	2.079		
	N	54	54	54	54	54		
	Significance vs. control	ns	ns	ns	ns	***		
Micafungin	Mean dL/L	8.033	7.791	7.796	9.436	10.01	10.71	
	SD	2.333	1.775	2.024	2.350	3.156	3.196	
	N	54	54	54	54	53	54	
	Significance vs. control	ns	ns	ns	*	***	***	
Fluconazole	Mean dL/L	10.14	10.55	10.28	9.784	9.518	*9.957*	*9.670*
	SD	2.636	1.990	2.588	2.709	2.585	*2.379*	*2.978*
	N	54	54	54	54	54	*54*	*54*
	Significance vs. control	ns	ns	ns	ns	ns	*ns*	*ns*

*Blank cells *are depicted where no measurements were possible, *italics* indicate differential treatment regarding fluconazole concentration (33 = 20 µg/ml and 100 µg/ml concentration was achieved by adding five times the volume of solution than other treatments)*ns* not significant*p ≤ 0.05, **p ≤ 0.001, ***p ≤ 0.0001

For all echinocandins the ratio of round-shaped, non-contracting versus rod-shaped normal-contracting cardiomyocytes increased dose-dependently. A stable rate of 44.3–53.5 % round-shaped cardiomyocytes was observed for anidulafungin concentrations from 0.1 to 10 μg/ml, whereas 86.6 % and 99.8 % of cardiomyocytes were round-shaped at 33 μg/ml and 100 μg/ml, respectively (Fig. 1 **c**; Tab. 2). Caspofungin concentrations of 0.1 and 1 μg/ml provided comparable proportions of rounded cardiomyocytes as controls while the proportion increased dose-dependently from 3.3 µg/ml to 100 µg/ml from 63.6 % to 73.1 %, 97.2 % and 100 %, respectively. At micafungin concentrations of 0.1–10 μg/ml equivalent fractions of round-shaped cardiomyocytes were seen as in controls; however, at concentrations of 33 and 100 μg/ml cardiomyocytes revealed a rounded shape in 66.4 and 95.3 %, respectively. Fluconazole showed no significant effect on shape at all analyzed concentrations.

**Tab. 2:** Values of cell morphology *(% rounded)*, standard deviation *(SD)* and number of cells *(N)* evaluated for all concentrations and preparations

		Concentration (µg/ml)						
		Control	0.1	1	3.3	10	33	100
Anidulafungin	% Rounded	48.1	44.3	53.5	49.7	47.3	86.6	99.8
	SD	13.0	12.0	12.0	7.0	9.0	9.0	1.0
	N	496	443	498	481	557	591	612
Caspofungin	% Rounded	49.7	44.1	54.0	63.6	73.1	97.2	100
	SD	11.5	12.0	17.9	17.5	9.0	3.3	–
	N	474	440	433	423	431	515	441
Micafungin	% Rounded	45.4	47.5	50.9	49.5	44.1	66.4	95.3
	SD	7.5	12.5	17.2	10.0	16.4	10.9	3.2
	N	495	491	526	492	474	476	476
Fluconazole	% Rounded	51.7	53.3	51.9	48.1	56.1	*55.2*	*49.4*
	SD	12.6	9.5	10.3	7.6	16.5	*8.9*	*13.7*
	N	403	426	481	430	474	*529*	*403*

Figures in *italics* indicate differential treatment regarding fluconazole concentration (33 = 20 µg/ml and 100 µg/ml concentration was achieved by adding five times the volume of solution than other treatments)

The rate of round-shaped cardiomyocytes, following high-dose echinocandins given in culture medium containing 10 mg/ml albumin, proceeded according to assumed concentration gradients (Fig. 1 **d**). Preincubation of echinocandins with 10 mg/ml albumin led to cancellation of all of the described effects.

## Discussion

The results revealed for the first time that echinocandin preparations in contrast to fluconazole have a dose-dependent impact on function and shape of isolated rat cardiomyocytes in vitro. Nevertheless, there has already been evidence of an ex vivo cardiotoxicity in rats performed with a Langendorff heart experiment [16]. Some very recent clinical reports described severe hemodynamic instability during anidulafungin administration in a critically ill ICU patient [17] and a flash pulmonary edema of unknown origin in a 52-year-old male [18]. Cardiac effects following echinocandin administration have also been seen in septic ICU patients [12]. Additionally, Stover et al. [16] summarized four cases from the FDA adverse events reporting system (FAERS) which may reflect the cardiac impact of the echinocandins: two reports of arrhythmia (anidulafungin), one cardiac failure (anidulafungin) and one described as sudden cardiac death (caspofungin; [19]).

Regarding effective tissue concentrations, there are some limits in transferring the effects of isolated cardiomyocytes to an in vivo situation. Cardiomyocytes are protected in vivo by an endothelial barrier; however, disrupted endothelial layers in sepsis might promote increased antifungal tissue concentrations. Consequently trough concentrations of caspofungin in critically ill patients were found to be higher (mean 2.16 µg/ml) than in healthy subjects [20]. Additionally, even in healthy patients caspofungin already reaches peak plasma concentrations up to 20 µg/ml [21] and 9.94 µg/ml in steady state, while heart tissue reaches about 50 % of plasma concentrations [22, 23] anidulafungin reaches maximum levels up to 14 µg/ml [24, 25] and the peak micafungin plasma concentration is approximately 8.8 µg/ml [26]. Recommended dose regimens provide loading dosages for caspofungin and anidulafungin which could lead to even higher intramyocardial concentrations. Regarding this, all clinical echinocandin effects which were reported in the case reports [12, 17] were seen according to initial loading dosages (200 mg anidulafungin and 70 mg caspofungin) in critically ill septic patients.

Because echinocandins are highly protein-bound in plasma and thus the total concentration could be many times higher than the free concentration, albumin was added to mimic plasma conditions. Primarily, it has to be considered that serum albumin levels are frequently decreased in patients at risk for invasive fungal infections which would increase the active drug concentration. Additionally, no data concerning the kinetics of protein binding of echinocandins following bolus infusion in humans appear to have been published. Especially in situations when antifungal treatment is needed, it is mostly administered through a central line, which could be followed by high active cardiac concentrations due to less time to bind to proteins. Moreover, standard minimum inhibitory concentrations (MIC) of echinocandins used to predict treatment response are estimated in the absence of albumin. However, albumin addition to this microbiological testing significantly raises the MIC to values above the cut-off that is applied to classify microbiological resistance. Data from phase 2 and 3 studies have not described adverse events regarding cardiac failure following echinocandin administration. Product information for caspofungin from the European Medicines Agency (EMA) mentions occasional congestive heart failure. However, most studies on antifungal agents did not include a relevant rate of critically ill patients and therefore, hemodynamic reserves might have been sufficient in the overall study populations. In contrast in septic patients it is possible that a drug-related cardiac failure is wrongly attributed to a progress of illness. According to the drug evaluation documents from EMA and the U.S. Food and Drug Administration (FDA), hypotension and a hemodynamic breakdown are described for an experimental administration of caspofungin in rats. This was associated with vascular histamine release. It has not yet been examined if histamine release is a possible mechanism of the observed effects. Nevertheless, the very recent report from Fink et al. [17] and in vivo observations in three patients [12] lead to the assumption that there must be an additional non-histamine-related pathway which leads to cardiac breakdown. Additionally histamine-releasing cells (e.g. mast cells) as mediators were not observed in the preparations in the current study.

The results of the study do not give the explanation for the mechanism of the observed effects on isolated cardiomyocytes by echinocandins. As Stover et al. [16] based on other publications [27, 28] already mentioned, there are several possible mechanisms of drug-induced cardiomyopathy. The evidence of myocyte and mitochondrial damage was observed using transmission electron microscopy [16]. Furthermore, the observed rounded shape of isolated cardiomyocytes in the current experiments indicates a disturbed intracellular calcium homeostasis. However, the possibility of a receptor mediated mechanism or direct toxic effects, e.g. due to oxidative stress cannot be ruled out. Because of the rapidity in which the effects appeared, alterations in cardiomyocyte gene expression or protein synthesis are not very likely to be the reason for the observed effect. Because of the absence of endothelial cells in the experimental set-up any involvement of endothelial cells in the observed results can be ruled out. Remarkably, lower doses of anidulafungin (3.3 and 10 µg/ml) and micafungin (3.3, 10 and 33 µg/ml) caused an increase in contractility responsiveness whereas caspofungin (10 µg/ml) led to a decrease. However, the same rounded fibrillating shape of the isolated cardiomyocytes without a straightened contraction was observed when exposed to high doses of all echinocandins. Although the molecular structure of the scaffold is very similar, the echinocandins have some differences in the side chains what is assumed to be the reason for such differences [25]. This might also be the reason why the MICs of the echinocandins are not equal [8, 29, 30]. Nevertheless, the rate of cardiomyocytes with a round cell shape consistently increases for all echinocandins dose-dependently.

There are some limitations of the study: First of all, the results from isolated rat cardiomyocytes are not transferable one-to-one to the human clinical setup on the ICU. Isolated cells are not protected by endothelium and higher free echinocandin expositions are even implicated by the absence of albumin in the experiment. Additionally, clinical case reports described effects in septic critically ill patients whereas the experiments were performed in cells harvested from healthy animals. Due to the experimental approach, it is not possible to give decisive recommendations for the clinical choice of any echinocandin preparation or a required modification of the way of administration. Finally, it is not clear whether the observed effects are selective for cardiomyocytes.

## Conclusion

Echinocandins impact the in vitro contractility of isolated cardiomyocytes of rats. This observation could be of great interest in the context of antifungal treatment. The setup used is experimental but gives an additional indication that echinocandins could have a potential impact on cardiac function. Further preclinical and clinical studies are necessary to evaluate the impact of echinocandin administration on different disease conditions, such as severe sepsis or septic shock.
